# The role of substance use treatment in reducing stigma after release from incarceration: A qualitative analysis

**DOI:** 10.1186/s40352-023-00225-w

**Published:** 2023-05-16

**Authors:** Kelly E. Moore, Janan P. Wyatt, Sarah Phillips, Catherine Burke, Chyrell Bellamy, Sherry A. McKee

**Affiliations:** 1 East Tennessee State University, 420 Rogers-Stout Hall, P.O. Box 70649, Johnson City, TN 37614 USA; 2 Yale University School of Medicine, 2 Church St. South, #109, New Haven, CT 06519 USA

**Keywords:** Stigma reduction, Self-stigma; substance use treatment, Reentry, Formerly incarcerated; peer support

## Abstract

**Background:**

People with substance use disorders (SUD) who have been involved in the legal system often experience stigma upon reentry into the community after incarceration. Although substance use treatment can sometimes be a source of stigma, it may also reduce stigma by facilitating connections with providers, reducing distress, or helping people feel more integrated in their community. However, research has rarely examined the potential for treatment to reduce stigma.

**Methods:**

This study examined stigma experiences and the degree to which substance use treatment reduced stigma among 24 people with SUDs who were receiving care in an outpatient treatment facility after release from incarceration. Qualitative interviews were conducted and analyzed using a content analysis approach.

**Results:**

Participants reported negative self-judgements as well as perceiving negative judgments from the community upon reentry. With regard to stigma reduction, themes emerged around substance use treatment repairing strained family relationships and reducing participants’ self-stigma. Aspects of treatment that reportedly reduced stigma included the treatment facility having a nonjudgmental atmosphere, patients trusting the staff, and working with peer navigators who had lived experience of SUD and incarceration.

**Conclusions:**

Results from this study suggest that substance use treatment has the potential to decrease the negative impacts of stigma upon release from incarceration, which continues to be a major barrier. Though more research on stigma reduction is needed, we suggest some preliminary considerations for treatment programs and providers.

More than 60% of people incarcerated in jails and 40% of people incarcerated in prisons have substance use disorders (SUDs; Bronson et al., 2017), making substance use one of the most common treatment needs among incarcerated populations. Indeed, substance use treatment is often identified as a priority for people reentering the community after incarceration (Morani et al., 2011), and is frequently mandated as a condition of probation/parole (Evans et al., 2009). However, many people struggle to successfully complete substance use treatment and remain out of legal trouble after release (Kopak et al., 2016; Lang & Belenko, 2000; Patra et al., 2010). Stigma associated with both addiction and legal involvement is one of the many barriers that people face as they reenter the community and initiate community-based substance use treatment (Keene et al., 2018; van Olphen et al., 2009). Expectations about being judged for having a history of SUD and legal system involvement can negatively impact self-worth, motivation for treatment, and broader integration in the community (Luoma et al., 2012; Moore et al., 2017). Although substance use treatment is sometimes thought to increase stigma via the application of a “disorder” label (i.e., Link & Phelan 1999), treatment may actually reduce stigma. Prosocial connections with therapeutic staff may reduce the degree to which a person feels judged by others in the community; also, general reductions in distress throughout therapy as well as connections with people who have lived experiences of SUD/incarceration may reduce shame and self-judgments (Snow et al., 2019; Latuskie et al., 2019). However, research has yet to explore the role that substance use treatment can play in reducing stigma among people recently released from incarceration. Given that stigma can negatively impact self-worth, reduce treatment motivation, and hinder integration in the community, thus increasing the likelihood of relapse and recidivism in this population (Moore et al., 2018; Newman et al., 2021), it is critical to understand how treatment may serve to reduce stigma.

## Defining stigma

Stigma broadly refers to a multifaceted process in which members of society devalue certain characteristics or attributes, and the interplay of structural and social disadvantages as well as social-cognitive impacts that people with these characteristics or attributes experience that impede their ability to live healthy lives (Link & Phelan, 2001). There are several theoretical conceptualizations describing how one experiences stigma; because this is a qualitative study, we do not constrain our definition to any one theoretical orientation and rather refer to a variety of stigma components and related factors, such as feeling judged, not trusting others, etc. Several stigma components that we highlight throughout are generally agreed upon in the literature, such that stigmatized people are aware of negative judgments about their identity (i.e., perceived stigma; Link et al.,2001) and these negative judgements are coped with in various ways, sometimes becoming internalized as accurate reflections of the self and causing negative cognitive/emotional states like shame and worthlessness (i.e., self-stigma; Corrigan et al., 2006). As a result of stigma experiences, people in stigmatized groups often feel disconnected from the broader community and discouraged from pursuing prosocial activities that could involve the potential for rejection (e.g., “why try” effect, Corrigan et al., 2009). Thus, stigma can present a barrier to achieving reentry or recovery goals after incarceration and it is important to understand how substance use treatment may or may not serve to reduce stigma.

## The stigma of substance use and legal involvement

People with SUDs and histories of incarceration, in particular, are among the most highly stigmatized in the U.S. due to the universal blame that is tied to these experiences. Common negative attitudes about this group include that they are dangerous, untrustworthy, unmotivated for treatment, lazy, immoral, and unable to change (Rade et al., 2016; Yang et al., 2017). In addition, many people believe substance use is a choice and a moral failing (Luoma et al., 2010; Wakeman et al., 2018) and therefore do not support rehabilitative approaches for incarcerated people (Hirschfield & Piquero, 2010). In addition to the judgment they face, people with SUD and legal histories are frequently rejected from housing, employment, and financial assistance, and often do not have access to other resources, such as familial support, that are necessary to maintain a stable, healthy lifestyle (Pager et al., 2009; Schnittker & John, 2007).

Within the substance use treatment context, stigma has been found to be especially problematic. It is well-known that people with SUDs often perceive and experience negative attitudes from the public that impact their beliefs about and engagement in substance use treatment (Crapanzano et al., 2019; Matsumoto et al., 2021). This is especially true for people with SUDs that are also involved in the legal system, who have reported feeling stigmatized and looked down upon by providers while in substance use treatment (van Olphen et al., 2009). Research has also shown that healthcare providers, including substance use treatment providers, hold negative attitudes about people with SUDs which diminish the quality of the treatment that they provide (van Boekel et al., 2013, Simon et al., 2020). In contrast, though people with SUDs and previous legal involvement face many stigma experiences, including during treatment, substance use treatment presents an opportunity to counter negative expectations and help build connections within the broader community.

## The potential for substance use treatment to reduce stigma

Research has rarely examined how substance use treatment may impact one’s experience with stigma, especially among people with histories of incarceration, and which elements of treatment are responsible for producing such changes. Some quantitative studies have shown no changes in substance use self-stigma over the course of treatment (Kulesza et al., 2014), while others have shown significant decreases in mental health self-stigma throughout treatment (Pearl et al., 2017). Moreover, qualitative studies have shown that participants explicitly mention lack of perceiving stigmatizing attitudes from others as a motivator that kept them engaged in substance use treatment and recovery (Latuskie et al., 2019; Snow et al., 2019).

Regarding aspects of treatment that may reduce perceived and/or self-stigma, staff characteristics such as compassion and a nonjudgmental stance have been identified as positive qualities of substance use treatment providers that make patients feel comfortable and accepted (Snow et al., 2019; Latuskie et al., 2019). In addition, feeling understood and being able to trust providers (Yang et al., 2018) have been identified as important for increasing patients’ comfort and engagement in substance use treatment. A strong therapeutic relationship with a treatment provider who is part of the broader community has the potential to provide a trusting relationship whilst challenging patients’ expectations about being judged by others. This is critical considering the strain that SUD places on relationships, particularly families (Orford et al., 2010), and the loss of trust that people with SUD report from others they may have previously relied on for social support (Smith et al., 2016).

Peer navigators with lived experience are also part of some treatment settings and may provide an additional layer of support that addresses self-stigma through normalizing people’s experience with SUD and incarceration. Peer navigators are thought to provide emotional, instrumental, informational, and affiliational support to people in substance use treatment (SAMHSA, 2009), and studies have found that patients appreciate being able to relate to people who have had similar experiences as they navigate treatment and reentry (Snow et al., 2019; Pantridge et al., 2016; Collins et al., 2019; Barrenger et al., 2019).

Finally, formerly incarcerated individuals reporting self-stigma score highly on measures of depression and low self-esteem (Moore et al., 2018); addressing these issues in treatment may also ameliorate negative cognitions and shame that are inherent in self-stigma. Several studies highlight improvements in self-esteem or self-efficacy as a major benefit of substance use treatment (Latuskie et al., 2019; Snow et al., 2019), and these general improvements throughout treatment likely decrease feelings of self-stigma. Despite these possible leads on aspects of treatment that could reduce stigma, research has not explored the connection between these positive elements of treatment to formerly incarcerated individuals’ experiences with stigma.

## Present study

Stigma can be problematic for people reentering the community after incarceration, especially as they integrate into community-based substance use treatment settings. However, substance use treatment has the potential to reduce stigma. This qualitative study aimed to better understand the stigma experiences people have as they are being released from incarceration and explore whether participation in a substance use treatment program reduces stigma experiences, including perceptions of how they are viewed by others (i.e., perceived stigma) as well as internalized feelings (i.e., self-stigma). This study also sought to explore which specific qualities of treatment assist in reducing experiences of stigma for this population. The present study was informed by the aforementioned literature review and seeks to answer the following research questions:

1. What types of stigma experiences are reported among people in treatment for substance use problems after release from incarceration?
2. To what extent does substance use treatment play a role, if any, in reducing stigma?
3. Which aspects of substance use treatment, if any, are reported to reduce stigma?

## Methods

### Participants and procedures

Participants in the present study included 24 adults who participated in a larger, federally-funded study evaluating a comprehensive care model of outpatient substance use treatment (McKee, 2021). Approval for this study was granted by the Yale School of Medicine Institutional Review Board and conducted in accordance with the Declaration of Helsinki. Incarcerated or recently released individuals were eligible to participate in the parent study if they showed evidence (i.e., based on their prison intake form) of substance use problems, but did not have serious mental illness (i.e., psychosis). All participants provided written consent to participate prior to engaging in research. The treatment clinic provided outpatient evidence-based individual weekly therapy and case management for a minimum of 12 weeks, following release from incarceration. Peer navigators facilitated treatment engagement, connections to medical and dental care, and led a weekly support group. Participants were evaluated by a psychiatrist and received medication management (e.g., psychiatric medications, medications for addiction treatment). Clinicians providing individual therapy included masters level counselors and pre- and postdoctoral trainees in clinical or counseling psychology. Urine toxicology screens were conducted weekly and staff communicated with the courts and community supervisors as appropriate to verify participants’ engagement and progress in the program, which was sometimes required as a condition of probation/parole.

Participants who engaged with the substance use treatment facility after release and successfully completed treatment (i.e., consistently attended for three months) were invited to complete a 45–60 min qualitative interview at the treatment facility, conducted by a researcher who had not met with the person clinically. The 24 participants in the present study were the first 24 to complete this qualitative interview after treatment. The interviews were audio-recorded and completed by a researcher experienced in conducting qualitative interviews. Participants were compensated with a $30 gift card for completing the interview. Participants were 42.3 years old on average (SD = 10.5) and 50% (n = 12) were women. With regard to race/ethnicity, 58.3% (n = 14) of participants identified as White/Caucasian, 29.2% (n = 6) identified as Black/African American, 8.3% (n = 2) identified as Hispanic/Latinx, and 4.2% (n = 1) identified as multi-racial. At the start of their treatment, four participants met criteria for cocaine use disorder, nine met criteria for alcohol use disorder, ten met criteria for opioid use disorder, and one met criteria for cannabis use disorder.

### Interview questions

Questions for this study were drawn from a larger qualitative interview guide that explored experiences about re-entering the community after incarceration as well as perceptions of the treatment program and their progress throughout treatment. Relevant to this study, we focused on several questions that asked about stigma experiences, including (1) During incarceration, how did you expect people to treat you upon returning to the community?, (2) Did you think anyone would discriminate against you because of your substance use or criminal record?, (3) How has that changed throughout the course of this program?, and (4) What does this program do to reduce stigma?

### Coding

We used a qualitative content analysis approach to analyze our data (Bengtsson et al., 2016), which has been described as a flexible qualitative approach that allows for inferences to be drawn from written or spoken text (Berelson, 1952; Krippendorff et al., 2004). Our qualitative analysis team included four researchers, all with doctoral degrees in clinical psychology, and two that had expertise in analyzing qualitative research. After the qualitative interviews were transcribed; a random subset of transcripts (n = 10) were reviewed, focusing on questions that asked specifically about stigma, to identify meaningful units and generate preliminary codes. These two coders reviewed and discussed the preliminary codes, subsequently sharing this information with the other two coders. With a preliminary coding frame in mind, the two coding teams each coded 12 full interview transcripts to identify and code all participant responses that related to the perception or experience of stigma. Once all transcripts were coded, meaningful units and codes were discussed between each team of coders, resolving discrepancies and consulting with a coder from the other team when resolution required a third coder’s perspective. When codes were finalized, the lead author reviewed all codes to propose a categorical framework for organizing the codes. This framework was discussed with all coders and revised as needed until agreement was reached. Each coding team then organized their transcript codes into the categories and cross-checked the other team’s categorization of codes. Themes were identified by reviewing the meaningful units, categories, and codes.

## Results

Findings from the individual interviews are organized by the three aforementioned research questions. For each research question major themes and sub-themes were identified. Themes are described below coupled with participant quotes and examples in Table 1; quotes represent a variety of participant perspectives.

**Table 1:** Research questions, themes, and example quotes

*Research Question 1: What types of stigma experiences are reported among people in treatment for substance use problems after release from incarceration?*			
Major Theme		Example Codes	Quote Examples
Negative perceptions from others		Labeled negatively (e.g., “bad apple”), people expect they will never change	“It’s like something I definitely don’t share with people…I think you’re definitely viewed differently so I try like not to let that be known to anybody.”
Lack of access to basic needs		Rejected by employers, problems obtaining housing	“You know it was so hard for me to get a job, and just because of my record, they will not hire me. I got so many interviews and just because of my record they say no.”
Strained interpersonal relationships		Loss of family trust, rejection by family members	“My kids were embarrassed and humiliated. I did all the right things, and now I’m incarcerated, and there’s a stigma.”
***Research Question 2: To what extent does substance use treatment play a role in reducing stigma?***			
Treatment engagement changes others’ perceptions	Treatment rebuilt family trust, improved parole officer perceptions		“You know my attitude has been better and people knowing that I’m doing it (treatment), particularly family, it’s been helpful- just knowing that I’m getting help.”
Treatment engagement reduces self-stigma	Treatment increased confidence, treatment reduced shame		“It (treatment) lets me know that I’m somebody, somebody great, and that it’s helped me look at a different outlook, don’t let my past dictate my present and future.”
***Research Question 3: Which aspects of substance use treatment are reported to reduce stigma?***			
Clinic environment	Respect from staff, friendly atmosphere		“They (staff/clinicians) look at us like we’re humans. They treat us like we’re humans. And when I come here, I don’t feel like an ex-con.”
Connection with peers/people with lived experience	Easy to relate, nonjudgmental		“Everybody here was really great…I never felt like anybody here like discriminated against me or judged me… and even just being able to talk to other people… that were incarcerated or like been around it, it’s like somebody else who knows like what it’s like.”
Treatment provider factors	Able to trust provider, provider was encouraging		“He’s (counselor) the best…and even though he didn’t go through the same things in life that I went through or done, he listened. And he didn’t judge. And that’s big for me.”

### Types of stigma experiences

The first research question sought to comprehensively explore and understand how participants describe experiences of stigma post-release. Three major themes consistently emerged as participants described their stigma experiences and the impact of stigma on their lives post-release: (a) negative perceptions from others, (b) lack of access to basic needs, and (c) strained interpersonal relationships.

### Negative perceptions from others

Many participants expressed anticipated and/or actual experiences of others viewing or treating them negatively due to their incarceration history or SUD. One participant expressed, “I feel like if I told somebody or (if) they knew, they would view me differently.” Many participants frequently mentioned that they don’t disclose this information, out of fear of being judged. “It’s something I definitely don’t share with people…I think you’re definitely viewed differently so I try not to let that be known to anybody.” When describing experiences of stigma, participants often identified negative labels that others might use to describe them. For example, a participant described their reasoning for not telling co-workers about their substance use history, stating, “The people at my job… they don’t know, and it sucks, because you are viewed a certain way like, either, a drug addict or like a trouble maker…” Another explained, “People look at you as an addict, that’s what you are, that’s what you’re always gonna be.” When discussing how histories of incarceration impact others perception of them, one participant said others often believe “that we’re criminals, that we’re just gonna keep on doin’ the same thing that we always do.” Many participants expressed frustration around experiencing others negative perceptions of them, especially as they attempt to demonstrate that their past does not define them. One stated,

They see once a criminal, always a criminal. Which is not true. We change. Some of us, most of us, we change. But they don’t see that yet, so we have to prove ourselves. And it’s not easy… you will have to prove yourself to your community that you’re a changed person.

### Lack of access to basic needs

When participants were asked to describe their stigma experiences, many disclosed difficulties in gaining employment and securing housing, specifically as it relates to their legal record and their status as a formerly incarcerated person. Many described these incidents and the frustration that arose from consistently being turned away.

Numerous participants expressed feeling as though they are not given a fair chance with employers due to their histories, stating, “It just seems like…jobs, a lot of, places don’t want to give you chances, especially if you’re convicted.” One participant described their experience of being able to successfully attain interviews, but then ultimately losing the offer of employment due to their history, explaining,

My criminal record always came back to hurt me. I would go through interviews and then …they would see okay, felony record, and then they’re like, “Oh, we’ll call you back”...that’s still the discrimination part.

Another described a similar experience, stating, “You know it was so hard for me to get a job, and just because of my record, they will not hire me. I got so many interviews and just because of my record they say no.” Another participant was even told that she wouldn’t be able to enter the health profession due to her history; she explained, “It was [my] dream was always to be a nurse…I was told that I couldn’t be a nurse because of my felony record.”

Related to histories of incarceration, other participants discussed the difficulty of securing housing. One stated, “Trying to find some place to… even [live]…in big cities, everywhere you look, finding an apartment was hard for me, cus they’re like, “No” (after the) background checks.” While most participants described experiences in which they were denied employment and housing, others explained how their histories of incarceration and substance use impacted their personal relationships.

### Strained interpersonal relationships

Many participants described how their histories of incarceration and substance use negatively impacted their interpersonal relationships, most notably with family members. Participants identified trust as a major reason for strained familial relationships. One participant explained, “… My mom and my dad (were) like, ‘Where are going… are you really just walkin’ to the store?’ … it takes like a long time for people to trust you again.” Another person stated, “Even though my family was supportive, I knew that they were upset with me, an’ it was gonna be a whole thing again for them to trust me…” In addition to rebuilding trust, other participants described how family members viewed and treated them differently upon release. One stated,

My immediate family were a little iffy when I came home because I was viewed as a criminal for a violation of probate. I didn’t go rob a bank, or you know do some crazy things, arson, sexual assault…but I was viewed a little differently.

In describing the relationship with their children upon release, one participant said, “My kids were embarrassed and humiliated. I did all the right things, and now I’m incarcerated, and there’s a stigma.” While many described strained familial relations, other participants reported discontinued communication or relationships altogether with family. One participant disclosed, “There was one time when I came home (and) my daughter wouldn’t talk to me.” When describing how relationships changed post-release, another participant expressed, “They’re different, like my mom and I don’t really talk. She told me I’ll never get sober, I’ll always be a drunk, I’ve ruined her life.”

Collectively, participants reported that their experiences of stigma post-release manifested in negative perceptions from others, difficulty securing employment and housing, and ruptured relationships. However, participants also described how engaging in substance use treatment impacted these stigma experiences during reentry.

### Does substance use treatment reduce stigma?

Most participants expressed that engaging in substance use treatment decreased experiences of stigma. Throughout the interview process, participants identified the ways in which treatment reduced stigma, which elucidated two themes about treatment: (a) engagement changes others’ perceptions, and (b) engagement reduces shame and increases self-forgiveness.

### Treatment changed perceptions

Participants described how their treatment status changed others’ perceptions of them, specifically family perceptions. One participant expressed how being in the treatment program and their success with recovery has reduced stigma, stating, “Well, since I’ve been in this program, I’ve been sober. I mean I haven’t really been discriminated against since I’ve been in the program as far [as] people or family.” Another participant described how being in treatment improved the trust he received from family members, stating, “My wife can trust me now, that she knows I’m not going to use the money for negative things. I’m doing good.” Another participant described the reaction their family has had toward their treatment engagement, explaining, “ My attitude has been better and people knowing that I’m doing it (treatment), particularly family, it’s been helpful- just knowing that I’m getting help.” In addition to the reduction of others’ stigma and improvement in their interpersonal relationships, participants also described the influences of treatment on their self-stigma.

### Treatment reduces self-stigma

While all participants held their treatment in high regard, a subset of them specifically spoke to how treatment engagement helped them acknowledge and reduce self-stigma. Participants spoke extensively about how their histories of substance use and incarceration negatively influenced their self-esteem and their overall perceptions of self. One participant expressed, “I was down on myself…, just because of the being in prison and the alcohol abuse, and I think what it comes down to is that’s just not me. I’m not that person.” Other participants spoke directly to the treatment process and how it was able to transform their perceptions of self. When discussing how treatment changed how they thought about themselves, one participant stated, “. I don’t look at myself as a convicted felon no more. I look at myself as an honest taxpaying resident.” Another participant reflected on internal changes that have occurred since engaging in treatment, stating, “Yeah, I don’t judge myself as (much)… I used to think I’m (was) a bad person.” Not only was treatment engagement described as a process that decreased stigma and shame, treatment also helped participants increase self-acceptance and self-forgiveness. One participant referred to the skills he learned in treatment that help emotion regulation and in prevent resumed substance use. He expressed,

It (treatment) has definitely changed my view on myself, and now I kinda feel like, alright well it might be tough, but I can do it, and I will get through this, and... I have the tools now to you know, get through it, or hopefully prevent it.

Lastly, participants explained that treatment reduced their self-stigma by increasing confidence, offering an alternative perspective on themselves and their future. One participant inspired by treatment said, “It (treatment) lets me know that I’m somebody, somebody great, and that it’s helped me look at a different outlook, don’t let my past dictate my present and future.” The above-mentioned participant quotes highlight how engagement in substance use treatment not only reduces perceived stigma from others, but just as importantly reduces self-stigma and increases self-efficacy.

### Which aspects of treatment reduce stigma?

The third research question sought to better understand the specific elements of substance use treatment that participants identified as involved in reducing stigma. Three major themes consistently emerged as participants described the aspects of treatment that reduced stigma: (a) clinic environment, (b) engaging with peers/people with lived experience, and (c) provider experiences.

### Clinical environment reduces stigma

The positive clinic environment and atmosphere repeatedly emerged as a major theme when participants described treatment experiences that reduced stigma. Many participants described the clinical space as “safe,” “friendly,” “non-judgmental” and spoke at length about how the clinical space made them feel “open” and “comfortable.” Participants emphasized the importance of the atmosphere and interaction with staff, explaining that these factors communicated a message of respect. One participant stated, “Like I said, they don’t look down on you. They show you the utmost respect… some people never had that.” Another participant described an ongoing supportive experience at the clinic, explaining,

It’s been nothing but positivity from when I opened the door and I’m greeted by (front desk staff member) to the time that I sit down with (clinician)… and from the time that I walk past another counselor or something like that, you just feel love.

Other participants spoke specifically about the importance of the clinical environment and interactions in the context of stigmatized identities. One participant expressed, “They (staff/clinicians) look at us like we’re humans. They treat us like we’re humans. And when I come here, I don’t feel like an ex-con.” Another participant also commented on the difference between this experience and previous treatment episodes, stating, “I feel like all counselors are the same where they’re judgmental and (here) you’re not just another number walking in the door. They (current staff) actually treated you like a human being. Like a person.” Even though this substance use treatment program is designed for people who are involved with the carceral system, participants spoke to the importance of not feeling as though the program is geared toward people who have a history of incarceration. One said, “I feel safe here… I don’t feel like anybody judges you, I just….nobody walks on pins and needles around me and but nobody’s ordering me what to do either, it’s like I’m a regular person…” Another participant stated,

I deserve as much (respect) as someone that wasn’t incarcerated, and it’s really not brought up. I really don’t think of this place as I’m here only cuz I was in jail, I don’t think of that at all, I think of it as you know therapy.

Overall, this theme highlights how vital a non-judgmental and welcoming atmosphere is when working with people with highly stigmatized identities. Participants repeatedly described the importance of feeling humanized when they presented to the clinic and that this warm reception allowed them to feel safe and comfortable enough to engage in treatment, thereby allowing them to challenge some of the self-stigma that they had been experiencing. While the first theme captures the importance of the clinical environment and interactions, participants further described the types of clinical interactions they valued most.

### Connection with peers reduces stigma

The second theme that emerged from this research question was the value of being able to connect with members of the clinical staff who have lived experience with substance use and incarceration. Participants explained how this experience further demonstrated a non-judgmental environment, while also offering a unique treatment characteristic that they had not previously experienced. One participant stated, “You offered me to talk to other people that were incarcerated, so again that’s a technique that no other facilities (offer).” One participant discussed the importance of being able to relate to peers with similar incarceration histories, explaining, “They (peers on staff), I think of been through some of the same situations maybe. So it’s easy to relate.” Another described the ease of feeling accepted and being able to talk to staff with lived experiences, stating,

Everybody here was really great…I never felt like anybody here discriminated against me or judged me… and even just being able to talk to other people… that were incarcerated or been around it, it’s like somebody else who knows what it’s like.

Participants described the importance of interacting with staff with lived experience and explained that it validated their reentry experiences and helped them feel more understood. One participant spoke specifically about a group led by peer navigators at the clinic that helped reduced stigma by exposing them to others in the same situation, reporting,

That group that they were doing, … for people that just got outta jail, cus then it’s nice sometimes to hear, like other people that just got home…they’re going through the same thing I’m going through.

The abovementioned quotes highlight the significant contributions of peers who were integrated into the clinical staff team and how their presence and client interactions assisted in reducing stigma in substance use treatment.

### Provider factors reduce stigma

When participants spoke about their treatment experiences and the specific aspects that reduced stigma, many discussed their relationship with their individual provider. Specifically, many participants identified the positive feelings that emanated from the relationships they had with their providers. One participant stated, “I can just be myself with (counselor), absolutely.” Another expressed a similar sentiment, explaining, “You guys make us feel comfortable and make (me) comfortable and I feel free to express myself.” Others described their providers as “sincere,” “attentive to (my) needs,” “always so understanding,” and “always supportive.” One participant described the pivotal role that her provider played in reducing her self-stigma, describing, “(Counselor name) helped me a lot getting my confidence back, getting my self-esteem…I had none before I was incarcerated.” Other participants discussed the importance of the provider relationship for processing past experiences with substance use and/or incarceration and the self-stigma related to their past experiences. One participant expressed the role her clinician played in her experience with self-stigma, reporting,

I can’t take back what they (participant’s kids) saw and I am having a lot of trouble with that. That’s what I work on with (counselors name), but she says…instead of beating myself up with the bad stuff, I gotta look at the good stuff…but I’m too overwhelmed with the guilt and the shame… I can’t seem to let it go.

Another participant spoke about the importance of his provider’s non-judgmental stance, stating, “He’s (counselor) the best…and even though he didn’t go through the same things in life that I went through or done, he listened. And he didn’t judge. And that’s big for me.” Many participants expressed the value of provider characteristics and some even identified how clinician support impacted their desire to remain abstinent. One interviewee explained,

I like having my breath not smell like alcohol. I like not smelling like a pound of weed when I come through the door. That’s cool. I like that. So I don’t want to let myself down….it’s like these people (providers) really got love for me. I can’t let them down.

## Discussion

This study sought to better understand experiences of stigma among people with SUD who were participating in substance use treatment after incarceration, and the impact of treatment on stigma reduction. Consistent with extant literature, participants reported a great deal of perceived and self-stigma related to their SUD and legal history. In addition, participants identified several aspects of treatment that reduced their perception of others’ stigma or their own self-stigma.

## Experiences with stigma

Many participants reported that stigma experiences directly interfered with accessing community resources such as housing and employment. Un- and underemployment in particular predict mental health problems as well as continued substance use, and are considered major sources of health inequity for people involved in the legal system (Kim et al., 2015; Nagelhout et al., 2017; Hatzenbueler et al., 2013). Many participants identified labels such as “felon”, “criminal”, and “addict” when asked to describe how others view them, and there was a general perception that others did not want to give them a chance to demonstrate positive behavioral change. These types of negative attitudes about SUD and legal involvement can negatively impact self-worth and self-efficacy, which, in turn, can significantly hinder accessing basic needs and repairing strained relationships during reentry. Research not only demonstrates that people with SUDs and legal histories experience perceived and self-stigma (Luoma et al., 2008; Moore et al., 2016; van Olphen et al., 2009), but also that these experiences impact legal outcomes and recovery, including community integration (Moore et al., 2017) and treatment engagement (Newman et al., 2021). Results from this study suggest a continued need to address stigma surrounding SUD and legal involvement at multiple levels, with the general public, families, treatment providers, and people with histories of incarceration.

## How does treatment reduce stigma?

Several themes emerged around the powerful impact that treatment can have on reducing stigma. In particular, shifts in family perceptions were noted as important benefits of treatment. Demonstrating to family that participants were engaged in treatment appeared to reduce family members’ negative attitudes toward participants and their past legal involvement, as well as help repair trust that had been harmed by participant behavior during active addiction. The value of family support for substance use treatment and recovery is not a new concept—existing treatment models have been known to focus on and draw support from family relationships, such as Community Reinforcement and Family Training (Meyers et al., 1998).

In addition to improving family perceptions and relationships, participants also described the improvements of their self-perceptions prompted by treatment engagement. They discussed the importance of treatment in shifting the view of themselves as they made progress toward their recovery and began to move past their legal problems. Thus, staying engaged in and progressing through treatment appears to counter negative self-perceptions and low self-efficacy. More research is needed to further explore the impact of substance use treatment on self-stigma.

Although engagement and progress in treatment helped improve self-perceptions and increase feelings of self-efficacy, initially engaging people in substance use treatment is notoriously challenging. Currently, there are standalone, often brief, interventions designed to reduce self-stigma tied to substance use (Livingston et al., 2012) and legal involvement (Moore et al., 2023), which explicitly attempt to reduce the tendency to avoid situations that may involve stigma experiences, including substance use treatment. These interventions are still being studied to understand how they help people overcome self-stigma, but they may be helpful to initially increase buy-in for treatment as a supplement to existing community-based treatment programs until the person experiences the full benefit of substance use treatment.

There was a general sense that treatment helped participants begin to heal from previous traumas or mental health problems that they had never dealt with alongside a qualified treatment provider. Aspects of the clinicians, peer navigators, and clinic environment that conveyed nonjudgmental attitudes, mutual respect, genuine empathy, and understanding of participants’ life were described as being important to participants. Participants described the treatment program as being different from others they had been to where they felt as if staff were simply there because they had to be there, not because they genuinely cared about the patients’ lives. This concept is not new; decades of research on therapist factors suggests the importance of elements such as warmth, support, validation, and encouragement in retaining people in treatment (Roos & Werbart, 2013; Cook et al., 2015). Moreover, research is demonstrating the benefits that people with lived experience can have for treatment engagement and retention (Berg et al., 2021; Tracy & Wallace, 2016). Our results suggest that these types of treatment elements are critically important for people involved in the legal system.

### Limitations

Although this study provides one of the only examinations of how substance use treatment can reduce stigma among people leaving incarceration, it is not without limitations. Participants were drawn from a grant-funded substance use treatment program implementing a novel, comprehensive system of care that included many unique elements not reflected in the majority of substance use treatment programs. Moreover, participants in this study reported being highly satisfied with this particular treatment program, which may reflect unique aspects of this program. Although this allows us to begin formulating best practice recommendations about stigma reduction in the context of substance use treatment, our results have limited generalizability to other substance use treatment programs. In addition, we used a general framework for asking about and interpreting stigma in this study, which was not comprehensive and neglected to include many important stigma experiences. For example, we primarily focused on perceived and self-stigma, but there are other important experiences that may have occurred with anticipated, enacted, or felt stigma. Finally, it is impossible to draw conclusions about whether the treatment elements that participants identified as being beneficial to them actually reduce stigma; experimental research that compares treatment outcomes between approaches emphasizing stigma reduction strategies to standard of care is needed to draw these sorts of conclusions. In general, these types of designs as well as quantitative assessment tools would allow researchers to understand whether incorporating stigma reduction strategies into substance use treatment programming would be beneficial.

## Conclusions

Stigma is a challenge for people as they transition into their communities after incarceration and engage in community-based substance use treatment. However, outpatient substance use treatment clinics can play an important role in reducing the stigma tied to substance use and incarceration. This was an exploratory first attempt to understand how substance use treatment can serve to reduce stigma experiences among people leaving incarceration. Although more research is needed, programs can begin to increase awareness around elements that may impact their clients’ stigma experiences. Clinics could consider whether there are benefits of offering peer support and individual (as opposed to group) therapy services, and could begin to evaluate the degree to which their staff are warm, welcoming, and non-judgmental through assessments and quality checks. Furthermore, this study underscores the importance of the therapeutic alliance, and how integral it is as an active treatment ingredient when providing services, especially to those with intersecting stigmatized identities. Incorporating such elements into outpatient clinics may not only facilitate patient engagement in treatment, but may have broader positive impacts on recovery and legal outcomes.

## Data Availability

Data are available on request due to privacy or other restrictions.
